# Impact of a population-based HPV vaccination program on cervical abnormalities: a data linkage study

**DOI:** 10.1186/1741-7015-11-227

**Published:** 2013-10-22

**Authors:** Dorota M Gertig, Julia ML Brotherton, Alison C Budd, Kelly Drennan, Genevieve Chappell, A Marion Saville

**Affiliations:** 1 VCS Inc, 265 Faraday St, Carlton, VIC 3053, Australia; 2 School of Population Health, University of Melbourne, Melbourne VIC, Australia; 3 Australian Institute of Health and Welfare, Bruce ACT, Australia

## Abstract

**Background:**

Australia was one of the first countries to introduce a publicly funded national human papillomavirus (HPV) vaccination program that commenced in April 2007, using the quadrivalent HPV vaccine targeting 12- to 13-year-old girls on an ongoing basis. Two-year catch-up programs were offered to 14- to 17- year-old girls in schools and 18- to 26-year-old women in community-based settings. We present data from the school-based program on population-level vaccine effectiveness against cervical abnormalities in Victoria, Australia.

**Methods:**

Data for women age-eligible for the HPV vaccination program were linked between the Victorian Cervical Cytology Registry and the National HPV Vaccination Program Register to create a cohort of screening women who were either vaccinated or unvaccinated. Entry into the cohort was 1 April 2007 or at first Pap test for women not already screening. Vaccine effectiveness (VE) and hazard ratios (HR) for cervical abnormalities by vaccination status between 1 April 2007 and 31 December 2011 were calculated using proportional hazards regression.

**Results:**

The study included 14,085 unvaccinated and 24,871 vaccinated women attending screening who were eligible for vaccination at school, 85.0% of whom had received three doses. Detection rates of histologically confirmed high-grade (HG) cervical abnormalities and high-grade cytology (HGC) were significantly lower for vaccinated women (any dose) (HG 4.8 per 1,000 person-years, HGC 11.9 per 1,000 person-years) compared with unvaccinated women (HG 6.4 per 1,000 person-years, HGC 15.3 per 1,000 person-years) HR 0.72 (95% CI 0.58 to 0.91) and HR 0.75 (95% CI 0.65 to 0.87), respectively. The HR for low-grade (LG) cytological abnormalities was 0.76 (95% CI 0.72 to 0.80). VE adjusted *a priori* for age at first screening, socioeconomic status and remoteness index, for women who were completely vaccinated, was greatest for CIN3+/AIS at 47.5% (95% CI 22.7 to 64.4) and 36.4% (95% CI 9.8 to 55.1) for women who received any dose of vaccine, and was negatively associated with age. For women who received only one or two doses of vaccine, HRs for HG histology were not significantly different from 1.0, although the number of outcomes was small.

**Conclusion:**

A population-based HPV vaccination program in schools significantly reduced cervical abnormalities for vaccinated women within five years of implementation, with the greatest vaccine effectiveness observed for the youngest women.

## Background

Randomized clinical trials have shown that prophylactic human papillomavirus (HPV) vaccines are highly effective at preventing infection with HPV types 16 and 18, which cause about 70 to 80% of cervical cancers worldwide [1,2] and in preventing related cervical abnormalities, which are the precursors to cervical cancer [3,4]. HPV16 and 18 are detected in about half of all high grade cervical intraepithelial lesions (CIN2/3), and in an even higher percentage of CIN2/3 diagnosed in women under 30 years of age [2,5-7]. Two vaccines are commercially available - a bivalent vaccine targeting HPV types 16 and 18 and a quadrivalent vaccine which also targets HPV types 6 and 11, responsible for 90% of genital warts. Types 16/18/6/11 have been detected in about 30% of low-grade cervical cytological abnormalities, reflecting that these low-grade lesions indicate acute HPV infection, which can be caused by any of the 40 genital HPV types [8]. The trials showed greatest efficacy for women naïve to HPV at the time of vaccination, as the vaccines do not have any therapeutic effect on targeted HPV types.

These vaccines have been widely adopted in high income countries since 2007 with most countries targeting girls prior to commencement of sexual activity, between 9 and 13 years of age, with varying catch-up programs for older adolescent girls [9]. Australia was one of the first countries to offer a national, publicly funded vaccination program, using a three-dose schedule of the quadrivalent vaccine Gardasil^©^. Commencing in April 2007, the Australian vaccination program targeted 12- and 13-year-old girls in schools and offered catch-up vaccination for 14- to 17-year-old girls in schools and 18- to 26-year-old women in community based settings.

Australia is in a unique position to evaluate the impact of the HPV vaccine at the population level. The recommended starting age for cervical screening is 18 years or 2 years after the onset of sexual activity, whichever is later, and thus screening already overlaps with the vaccination program. Screening is recommended every two years using conventional cytology until the age of 69 years. Australia’s State based Pap test registers systematically record cervical cytology and histology results and send reminder and follow-up letters to women and their practitioners according to national guidelines. Participation in both screening and vaccination programs is high: catch-up HPV vaccine coverage in Victorian females 12 to 17-years old was 86/82/75% for doses 1/2/3 (JMLB personal communication) and 77% of 20 to 24-year-olds attended at least one screening between 2007 and 2011 [10].

In this retrospective cohort study, we linked data from the Victorian Cervical Cytology Registry (VCCR) and the National HPV Vaccination Program Register (NHVPR) and evaluated the effectiveness of the HPV vaccine against cervical abnormalities in a screening population of women eligible for vaccination in the school-based cohorts (aged 17 or younger in 2007). We focus on this cohort for two reasons: (1) effectiveness in this age group will most closely approximate the eventual impact of vaccination in the ongoing target group of girls prior to sexual activity and (2) vaccination status in the school cohorts is more completely notified than in the adult women catch-up program, where notification was not compulsory and under-notification has been estimated at 10 to 15% [11].

## Methods

### Data collection

The VCCR captures cervical screening results for all women resident in Victoria, Australia, which has a population of more than 2.7 million women, and receives data on cervical cytology and histopathology directly from laboratories in a timely manner. Fewer than 1% of women request to opt-off the VCCR [10]. Details regarding the information held on the VCCR have been published previously [10]. The VCCR supports the national screening policy by sending reminders and contacting women and their practitioners when results of indicated follow-up tests are not received by the register. Australian guidelines recommend referral to colposcopy for all women with a high-grade abnormality suspected or diagnosed on cytology and for all glandular abnormalities. It is recommended that women with low grade cytology have a repeat Pap smear at 12 months, except in women over 30 years of age with an inadequate screening history or when two consecutive low-grade abnormalities have been reported [12].

The NHVPR was established in June 2008 and records HPV vaccination doses administered nationwide. Details on the establishment of the NHVPR, and initial coverage in both the schools program and the catch-up program have been published elsewhere [11,13,14]. In brief, doses administered through schools are received from school immunization providers, which are predominantly local councils and health departments, and notification from these providers is believed to be virtually complete. Doses administered in the school programs prior to register establishment were also systematically uploaded to the register.

### Data linkage

Cross-referencing of data against cervical cytology registers for monitoring of the effectiveness of HPV vaccine is allowed for by legislation enabling operation of the NHVPR. However, because the VCCR was established far earlier, there is no similar provision for the VCCR and thus individual identifiers could not be used for the data linkage in this study.

**Figure 1:**
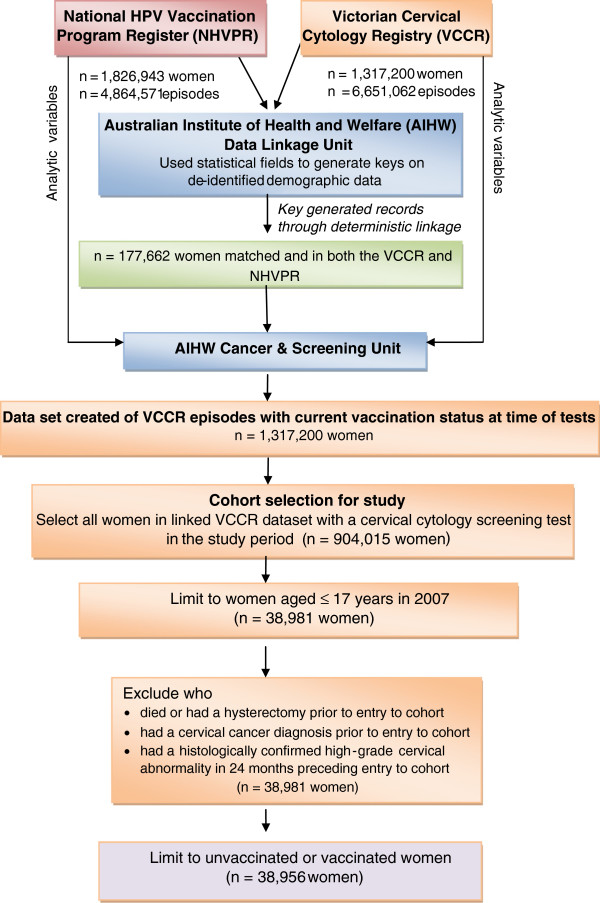
Data linkage process and exclusions for analysis.

A deterministic linkage procedure was undertaken to link records from the VCCR, which included Victorian women who are age-eligible for the HPV vaccine as per product indication (date of birth on or after 1 July 1962), to the NHVPR which included all Australian women who received the HPV vaccine prior to 1 January 2012 (Figure 1).

At the time of extraction, data from the VCCR and NHVPR were de-identified in the same manner. Randomly generated record ID numbers were assigned to women in each register. The Data Linkage Unit generated a series of 22 linkage keys using combinations of variables, such as selected letters from given name and surname, perturbed date of birth, postcode and parts of the Medicare number. The quality of these linkage keys was assessed by calculating the percentage of unique combinations each would be likely to produce. The first 16 linkage keys produced a set of unique combinations with the best balance between incorrectly accepting a given record pair as the same person versus failing to accept a given record pair as the same person, and these were used to link records in a deterministic manner.

### Study design and inclusion criteria

A retrospective cohort was constructed of women aged 17 or younger in 2007 who had a Pap test recorded on the VCCR during the study period, 1 April 2007 (the date the HPV vaccination program commenced) to 31 December 2011. Women were counted as at risk of a diagnosis of a cervical abnormality from the time they commenced cervical screening, and were entered into the cohort at their first Pap test (or on 1 April 2007 if their first Pap test was prior to that time). We found that our analyses were robust when the entry criteria to the cohort for those screening prior to April 2007 were altered to include person-time from their first Pap test after 1 April 2007. Women were followed until the outcome of interest, date of death, hysterectomy or the end of the study period.

### Assignment of vaccination status

Unvaccinated women were those who had no doses of HPV vaccine recorded on the NHVPR; vaccinated women were those who received any doses of HPV vaccine. Completely vaccinated women were those vaccinated with three doses compliant with the vaccine schedule (and, if doses were closer than recommended minimum intervals, where no further doses were recommended according to Chief Medical Officer guidelines. Women with further doses needed according to the guidelines were excluded from the analyses (n = 25) [15]. Person-time and events were allocated to each “woman-dose” according to her vaccination dates. For unvaccinated women, person-time commenced from the date of first Pap test as above. For vaccinated women, a new vaccination status (number of doses) was assigned on the date of that dose and person-time at that dose number was included until receipt of a further dose (if any). Where screening and vaccination occurred on the same day, if a screening-related outcome was diagnosed on that test, the woman’s vaccination status for that outcome was assigned to her vaccination status during the period immediately prior to that date (dose delivered -1); hence, prevalent abnormalities were assigned to the relevant vaccination status.

We chose the dose assignment method, with no time-lag until abnormalities are ‘counted’ after vaccination, because we wanted to reflect apparent incident abnormalities as they occurred in the real world (that is, what the patient and clinician would experience after vaccination as a measure of vaccine effectiveness). However, we recognize that abnormalities diagnosed during the vaccination course are predominantly caused by HPV infection acquired prior to the onset of vaccination. Therefore, we also evaluated the cohort using a measure closer to vaccine efficacy assessment, excluding time for vaccinated women where they were in the process of being vaccinated, that is, censoring time occurring between dose 1 and dose 3. This second method (presented in the Additional file 1: Table S1) counts person-time according to the woman’s final vaccination status and thus only includes person-time for partial vaccination where this was the woman’s final vaccination status. This will more closely reflect the actual efficacy of receipt of only one or two doses.

### Definition of outcomes

The primary outcome was histologically confirmed high-grade (HG) cervical disease (CIN2+/AIS), defined as CIN2, CIN3, adenocarcinoma *in situ* or mixed CIN3/AIS. Histological and cytological outcomes were assigned according to categorization used by the AIHW [12] and Australian Standardised Modified Bethesda System, respectively [16]. For all outcomes, a woman’s first relevant abnormality or her first in two years with at least two negative cytology tests in between, were counted. Abnormalities diagnosed on the first test were included as incident abnormalities.

### Statistical methods

Analyses of demographic and exposure characteristics of women in the cohort by vaccination status used the Mann-Whitney *U* test for ordinal variables and the Pearson chi-square test for nominal variables. Detection rates were calculated as the number of events per 1,000 person-years at risk and vaccine effectiveness was (1-rate ratio) × 100.

Cox proportional hazards regression was used to estimate hazard ratios (HR) and 95% confidence intervals (CI), and included *a priori* the other predictors: age at first screening (as a proxy for age at sexual debut), socioeconomic status and remoteness. Remoteness areas were assigned based on postcode of residence and as defined according to the Australian Standard Geographic Classification. Socioeconomic status was assigned by postcode of residence according to the Australian Bureau of Statistic’s Socio-Economic Indexes for Areas (SEIFA) Index of Relative Socio-Economic Disadvantage [17]. Lower socioeconomic status is associated with poorer screening participation and higher cervical cancer incidence in Australia [12]. Individuals were observed until detection of outcome or 31 December 2011, whichever occurred first. All data were analyzed using the Proportional hazards regression (PHREG) procedure using SAS Institute Inc software, Cary, NC, USA [18].

Ethics approval was obtained from the Department of Health and Ageing and the Australian Institute of Health and Welfare’s Human Research and Ethics Committees. Approval for use of NHVPR data was given by the Department of Health and Ageing, the data custodian and for the VCCR data by the Victorian Department of Health.

## Results

Between 1 April 2007 and 31 December 2011, 24,871 women aged between 12 and 17 years who were vaccinated against HPV had commenced cervical screening. Of these women, 21,151 (85.0%) were completely vaccinated and 3,690 women had received one or two doses of vaccine. There were 14,085 unvaccinated women of the same age who had commenced cervical screening. The follow-up period was a maximum of 4.8 years with an average of 1.5 years for both vaccinated women and unvaccinated women. There was no difference in mean age at entry into the cohort or mean age at first screen between vaccinated and unvaccinated women, with almost all first Pap tests occurring after April 2007, Table 1. Because the youngest girls at the time of vaccination were only just becoming eligible for cervical screening at the time of analysis, most women in the study were vaccinated between 14 and 17 years of age, with a mean age at vaccination of 15.7 years. Seventy five percent of women in this analysis who completed the vaccine course received their first dose in 2007, at the commencement of the Australian HPV vaccination program, with 20% commencing in 2008 (predominantly those aged 14 years and under in 2007). Unvaccinated women were more likely to be from a major city and of lower socioeconomic status (SES) than vaccinated women. Partially vaccinated women (one or two doses) were younger at first screen than either unvaccinated women or vaccinated women, much older at vaccine commencement and of lower socioeconomic status than completely vaccinated women (*P* <0.05).

**Table 1:** Summary of descriptive characteristics of cohort

	**Unvaccinated**	**Vaccinated, Any dose**	**Vaccinated, 1 dose**	**Vaccinated, 2 doses**	**Completely vaccinated, 3 doses**
**Number of observations**	14,085	24,871	1,422	2,268	21,151
**Mean age in 2007**	16.1 (± 1.0)	16.0 (± 1.0)^*^	16.0 (± 1.0)	16.0 (± 1.0)^#^	16.0 (± 1.0)
**Mean age at first screen**	18.5 (± 1.4)	18.5 (± 1.3)^#^	18.1 (± 1.5)^#^	18.1 (± 1.5)^#^	18.6 (± 1.3)
**Mean age at entry to cohort**	19.0 (± 1.4)	19.0 (± 1.3)	18.7 (± 1.3)^#^	18.7 (± 1.3)^#^	19.0 (± 1.2)^#^
**Age in 2007 (years)**					
≤14	1,446 (10.3%)	2,567 (10.3%)	163 (11.5%)	271 (11.9%)^*^	2,131 (10.1%)
15	2,434 (17.3%)	4,480 (18.0%)	262 (18.4%)	470 (20.7%)^#^	3,743 (17.7%)
16	4,125 (29.3%)	7,419 (29.8%)	372 (26.2%)^+^	628 (27.7%)	6,413 (30.3%)^*^
17	6,080 (43.2%)	10,405 (41.8%)^+^	625 (44.0%)	899 (39.6%)^+^	8,864 (41.9%)^*^
**Remoteness area**^**3**^					
Major cities	10,019 (71.8%)	16,608 (66.8%)^#^	955 (67.2%)^#^	1,477 (65.1%)^#^	14,154 (67.0%)^#^
Inner regional	3,250 (23.3%)	6,858 (27.6%)^#^	383 (26.9%)^#^	642 (28.3%)^#^	5,826 (27.6%)^#^
Outer regional	681 (4.9%)	1,380 (5.6%)^+^	83 (5.8%)	147 (6.5%)^#^	1,149 (5.4%)^+^
Remote	6 (0.0%)	14 (0.1%)	1 (0.1%)	2 (0.1%)	11 (0.1%)
**Socioeconomic status**^**4**^					
1 (lowest)	2,551 (18.4%)	3,830 (15.5%)^#^	278 (19.6%)	401 (17.7%)	3,147 (14.9%)^#^
2	2,833 (20.4%)	4,741 (19.1%)^+^	336 (23.7%)^+^	497 (22.0%)^*^	3,899 (18.5%)^#^
3	2,713 (19.6%)	4,463 (18.0%)^#^	262 (18.5%)	444 (19.7%)	3,754 (17.8%)^#^
4	3,195 (23.1%)	6,340 (25.6%)^#^	302 (21.3%)	572 25.3%)^+^	5,461 (25.9%)^#^
5 (highest)	2,571 (18.6%)	5,399 (21.8%)^#^	240 (16.9%)	346 (15.3%)^#^	4,804 (22.8%)^#^
**Age at first screen (years)**					
≤14	105 (0.7%)	114 (0.5%)^#^	13 (0.9%)	30 (1.3%)^+^	71 (0.3%)^#^
15 to 17	2,831 (20.1%)	4,840 (19.5%)	436 (30.7%)^#^	624 (27.5%)^#^	3,775 (17.8%)^#^
18+	11,149 (79.2%)	19,917 (80.1%)^*^	973 (68.4%)^#^	1,614 (71.2%)^#^	17,305 (81.8%)^#^
**Screening history**					
Screening before 1 April 2007	244 (1.7%)	385 (1.5%)	52 (3.7%)^#^	74 (3.3%)^#^	257 (1.2%)^#^
First screen after 1 April 2007	13,841 (98.3%)	24,486 (98.5%)	1,370 (96.3%)^#^	2,194 (96.7%)^#^	20,894 (98.8%)^#^
**Age commenced vaccination (years)**					
≤13		591 (2.4%)	19 (1.3%)^!^	63 (2.8%)	509 (2.4%)
14 to 15		9,609 (38.6%)	318 (22.4%)^$^	756 (33.3%)^$^	8,526 (40.3%)
16 to 17		13,519 (54.4%)	679 (47.7%)^$^	1,159 (51.1%)^$^	11,665 (55.2%)
18+		1,152 (4.6%)	406 (28.6%)^$^	290 (12.8%)^$^	451 (2.1%)
**Year entered cohort**					
2007	632 (4.5%)	497 (2.0%)^#^	46 (3.2%)^*^	52 (2.3%)^#^	398 (1.9%)^#^
2008	1,114 (7.9%)	2,140 (8.6%)^*^	188 (13.2%)^#^	248 (10.9%)^#^	1,701 (8.0%)
2009	2,350 (16.7%)	4,924 (19.8%)^#^	357 (25.1%)^#^	577 (25.4%)^#^	3,982 (18.8%)^#^
2010	3,981 (28.3%)	7,375 (29.7%)^+^	403 (28.3%)	607 (26.8%)	6,361 (30.1%)^#^
2011	6,008 (42.7%)	9,935 (39.9%)^#^	428 (30.1%)^#^	784 (34.6%)^#^	8,709 (41.2%)^+^
**Cytological abnormalities diagnosed on entry into cohort**^**5**^					
Negative	11,444 (82.7%)	19,661 (85.4%)^#^	911 (80.5%)^#^	1,579 (84.1%)^#^	17,148 (85.7%)
Low-grade	1,817 (13.1%)	2,488 (10.8%)^#^	169 (14.9%)	230 (12.2%)^#^	2,086 (10.4%)^#^
High-grade					
Possible	111 (0.8%)	146 (0.7%)^*^	8 (0.7%)	15 (0.8%)	123 (0.6%)^*^
Definite	97 (0.7%)	132 (0.6%)^*^	12 (1.1%)	12 (0.6%)	108 (0.5%)^*^
Endocervical	1 (0.0%)	4 (0.0%)	0 (0.0%)	0 (0.0%)	4 (0.0%)

^1^Count is of women; “unvaccinated” refers to women screened who did not receive any dose of HPV vaccine; “completely vaccinated” refers to women who were clinically completely vaccinated with three doses of HPV vaccine.

^2^Entry into the cohort was at first Pap test, or 1 April 2007 if screened before this date.

^3^Women were allocated to a remoteness area based on their postcode of usual residence, according to the Australian Standard Geographic Classification (ASGC) for 2006. Missing data on 140 women excluded.

^4^Women were allocated to a socioeconomic status (SES) groups based on their postcode of usual residence, according to the Australian Bureau of Statistics Socio-Economic Indexes for Areas (SEIFA) Index of Relative Socio-Economic Disadvantage [17]. Missing data on 320 women excluded.

^5^Missing data on 2,079 women excluded.

^*^P-value *P* ≤0.05 (reference group unvaccinated women).

^+^*P*-value ≤0.01 (reference group unvaccinated women).

^#^*P*-value ≤0.001 (reference group unvaccinated women).

^$^*P*-value ≤0.001 (reference group fully vaccinated women). Note any dose group not compared.

^!^*P*-value ≤0.01 (reference group fully vaccinated women). Note any dose group not compared.

The number and rate of cervical abnormalities detected by vaccination status as per our primary analysis (dose assignment method for assessing vaccine effectiveness) is shown in Table 2. A lower risk of any histologically confirmed HG cervical abnormality was observed for vaccinated women (any dose) compared with unvaccinated women with a hazard ratio of 0.72 (95% CI 0.58 to 0.91), after adjusting for age at first screening, SES and remoteness. This effect was strongest for completely vaccinated women; there was no significant reduction among those partially vaccinated (Table 2), but the number of outcomes was small. Similarly, detection rates of CIN3/AIS and CIN2+ were significantly lower for vaccinated women compared to unvaccinated women during the study period. There was a reduced risk of LG cytological abnormalities for women who received one or two doses of vaccine HR 0.66 (95% CI 0.60 to 0.72) compared with unvaccinated women. However, this result was sensitive to the censoring of time during the vaccination course for fully vaccinated women using our secondary analytic method (more similar to trial efficacy calculations) (see Additional file 1: Table S1). There were only three women in this cohort with outcomes of AIS, one occurred in a vaccinated woman and two in unvaccinated women.

**Table 2:** Number and rate of cervical abnormalities for completely vaccinated, partially vaccinated and unvaccinated women

**Outcome**		**No. women-doses**	**No. abnormalities**	Rate*	**Hazard ratio**
**Histological abnormalities**					
Any high grade	Unvaccinated	15,192	138	6.4	1.0
	Vaccinated (unadjusted)				0.76 (0.61 to 0.95)
	Vaccinated (adjusted)	27,179	181	4.8	0.72 (0.58 to 0.91)
	*1 dose*	*2,568*	*27*	*9.7*	*1.47 (0.97 to 2.23)*
	*2 doses*	*3,412*	*28*	*6.8*	*1.02 (0.68 to 1.53)*
	*1 or 2 doses*	*5,980*	*55*	*8.0*	*1.20 (0.88 to 1.65)*
	*Complete*	*21,199*	*126*	*4.1*	*0.61 (0.48 to 0.78)*
CIN3/AIS	Unvaccinated	15,192	61	2.8	1.0
	Vaccinated (unadjusted)				0.68 (0.48 to 0.95)
	Vaccinated (adjusted)	27,179	70	1.9	0.64 (0.45 to 0.90)
	*1 dose*	2,568	12	4.3	1.40 (0.75 to 2.61)
	*2 doses*	3,412	11	2.7	0.87 (0.46 to 1.67)
	*1 or 2 doses*	5,980	23	3.3	1.09 (0.67 to 1.76)
	*Complete*	21,199	47	1.5	0.53 (0.36 to 0.77)
CIN2	Unvaccinated	15,192	87	4.0	1.0
	Vaccinated (unadjusted)				0.81 (0.61 to 1.06)
	Vaccinated (adjusted)	27,179	122	3.2	0.78 (0.59 to 1.03)
	*1 dose*	*2,568*	*16*	*5.7*	*1.29 (0.76 to 2.20)*
	*2 doses*	*3,412*	*18*	*4.4*	*0.99 (0.59 to 1.64)*
	*1 or 2 doses*	*5,980*	*34*	*4.9*	*1.11 (0.75 to 1.66)*
	*Complete*	*21,199*	*88*	*2.9*	*0.70 (0.52 to 0.94)*
CIN1	Unvaccinated	15,192	163	7.5	1.0
	Vaccinated (unadjusted)				0.86 (0.70 to 1.05)
	Vaccinated (adjusted)	27,179	244	6.5	0.83 (0.68 to 1.02)
	*1 dose*	*2,568*	*20*	*7.2*	*0.89 (0.56 to 1.41)*
	*2 doses*	*3,412*	*30*	*7.3*	*0.90 (0.61 to 1.33)*
	*1 or 2 doses*	*5,980*	*50*	*7.2*	*0.90 (0.65 to 1.23)*
	*Complete*	*21,199*	*194*	*6.3*	*0.82 (0.66 to 1.01)*
**Cytological abnormalities**					
High-grade cytology	Unvaccinated	15,192	325	15.3	1.0
	Vaccinated (unadjusted)				0.77 (0.67 to 0.89)
	Vaccinated (adjusted)	27,179	442	11.9	0.75 (0.65 to 0.87)
	*1 dose*	*2,568*	*44*	*16.0*	*0.85 (0.62 to 1.17)*
	*2 doses*	*3,412*	*67*	*16.5*	*0.95 (0.73 to 1.23)*
	*1 or 2 doses*	*5,980*	*111*	*16.3*	*0.91 (0.73 to 1.13)*
	*Complete*	*21199*	*331*	*10.9*	*0.71 (0.61 to 0.83)*
Low-grade cytology	Unvaccinated	15,192	2,306	125.8	1.0
	Vaccinated (unadjusted)				0.77 (0.73 to 0.82)
	Vaccinated (adjusted)	27,179	3,184	95.3	0.76 (0.72 to 0.80)
	*1 dose*	*2,568*	*250*	*101.9*	*0.67 (0.59 to 0.76)*
	*2 doses*	*3,412*	*328*	*89.2*	*0.64 (0.57 to 0.72)*
	*1 or 2 doses*	*5,980*	*578*	*94.3*	*0.66 (0.60 to 0.72)*
	*Complete*	*21,199*	*2,606*	*95.5*	*0.79 (0.75 to 0.84)*

*Rate per 1,000 person-years.

All high grade histology defined as CIN2, CIN3, AIS and mixed CIN3/AIS.

High-grade cytology defined as possible high-grade squamous intraepithelial lesion (HSIL), HSIL, HSIL with possible microinvasion/invasion, squamous cell carcinoma, possible high-grade endocervical glandular lesion, AIS, AIS with possible microinvasion/invasion and adenocarcinoma.

Low-grade cytology defined as possible low-grade squamous intraepithelial lesions (LSIL), LSIL and atypical endocervical cells of uncertain significance.

Unvaccinated refers to women screened who did not receive any dose of HPV vaccine; completely vaccinated refers to women who were clinically completely vaccinated with three doses of HPV vaccine.

Hazard ratios adjusted for remoteness, SES and age at first screening.

**Figure 2:**
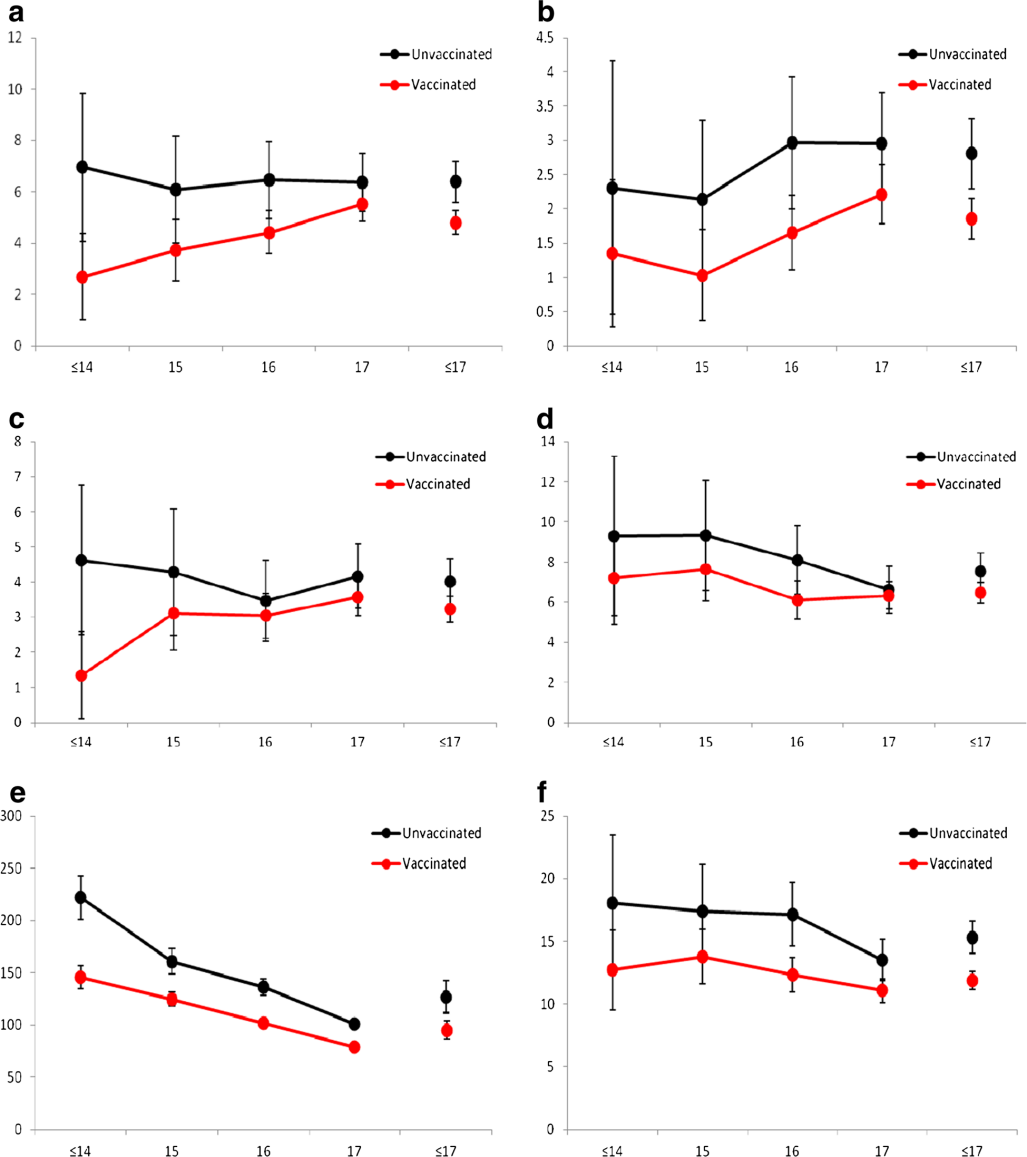
**Cervical abnormalities rates for vaccinated and unvaccinated women, by age in 2007.** High-grade histology per 1,000 women years, by age in 2007. **(a) (b)** CIN 3/AIS histology per 1,000 women years, by age in 2007. **(c)** CIN 2 histology per 1,000 women years, by age in 2007. **(d)** CIN I histology per 1,000 women years, by age in 2007. **(e)** Low-grade cytology per 1,000 women years, by age in 2007. **(f)** High grade cytology per 1,000 women years, by age in 2007. All high grade histology is defined as CIN2, CIN3, AIS and mixed CIN3/AIS. Age is presented in years.

Figure 2a-f shows the detection rates by age in 2007 and vaccination status for each outcome of interest. Detection rates for any histological outcomes increased with age and were lower for women who had received any dose of HPV vaccine compared with unvaccinated women at all ages. For cytological outcomes, rates were also lower for vaccinated women at all ages as compared with unvaccinated women for LG cytology. There was a suggestion of a reduced risk of HG histology for younger women who received one or two doses of vaccine rather than none as compared with older women but the numbers were small (data not presented).

**Figure 3:**
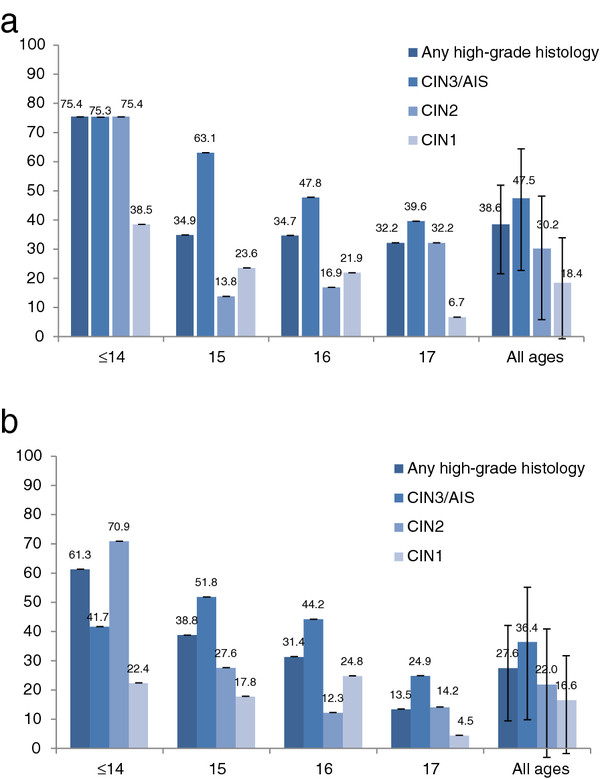
**HPV vaccine effectiveness for cervical histological outcome, by age in 2007, for (a) completed vaccine course, for (b) any vaccine dose.** All high-grade histology is defined as CIN2, CIN3, AIS and mixed CIN3/AIS. Vaccine effectiveness is defined as (1-adjusted hazard rate) x 100. Age in years, as of 2007.

Vaccine effectiveness (VE), adjusted for remoteness, SES and age at first Pap test, was highest for CIN3/AIS at 47.5% (95% CI 22.7 to 64.4) for women who were completely vaccinated (compared to no doses), (Figure 3a) and was slightly lower for women who received any dose of vaccine 36.4% (95% CI 9.8 to 55.1), Figure 3b). VE for histological outcomes was highest in younger women (that is, those who were less likely to be sexually active before vaccination) declining thereafter with increasing age. For most age groups, VE was lowest for CIN1 outcomes, although it is important to note that in Australia only a small proportion of LG cytology lesions lead to further investigation [16]. For HG cytology, adjusted VE for all ages combined for any dose of vaccine was 24.9% (95% CI 13.3 to 35.0), similar to that for LG cytology, adjusted VE 23.9% (95% CI 19.6 to 27.9).

## Discussion

Our results show that quadrivalent HPV vaccination substantially reduced cervical abnormalities for women receiving any HPV vaccine dose through a school-based program, within five years of implementation. Vaccine effectiveness against high-grade histological disease was higher than that observed in the intent-to-treat (ITT) analyses of the clinical trials of the quadrivalent HPV vaccine, which vaccinated women between the ages of 16 and 25, and was highest in younger women, who as a group are least likely to have been sexually active prior to vaccination. Vaccine effectiveness was greatest for outcomes most strongly associated with vaccine related HPV types 16 and 18, in particular CIN3/AIS, and lowest for low-grade cytological abnormalities for which the majority of disease is accounted for by non-vaccine HPV types. The reduction in risk of cervical abnormalities was restricted to women who received three doses of vaccine.

The follow-up time in our study was five years and further follow-up will likely impact upon the effectiveness of the vaccine. Our data confirm that VE diminishes with age of administration in catch-up programs, which is a proxy for the onset of sexual activity. The ongoing vaccination programs in Australia and elsewhere target girls prior to onset of sexual debut and at the population level higher reductions will be observed as this large cohort reaches screening age, as a greater proportion of vaccinated women will have been unexposed to HPV at the time of vaccination. The VE was higher in the youngest women in our study than observed in the ITT analyses of either the quadrivalent or bivalent vaccine trials [19-21]. The VE from ITT analyses, at an average of 3.6 years follow-up, for the quadrivalent vaccine irrespective of HPV type was 16.4% 95% CI (0.4 to 30.0), rate reduction 0.2 for CIN3, compared with 45.3% (95% CI 29.8 to 57.6), rate reduction 0.3, for the analyses restricted to disease related to vaccine types [3,22]. For the bivalent vaccine, the VE for CIN3 at four years end of study follow-up was similar for TVC analyses related to HPV 16 and 18 endpoints 45.7% (95% CI 22.9 to 62.2), rate reduction 0.13, as compared with endpoints irrespective of HPV type VE 45.6% (95% CI 28.8 to 58.7), rate reduction 0.22 [20].

Studies have shown that a greater proportion of CIN3+ lesions in younger women (under 30 years of age) is due to HPV type 16, with other less oncogenic types more prevalent in women over 30 years of age [5-7]. HPV16 is likely to overwhelmingly predominate as a cause of high-grade disease in women aged under 20, which is the age group at screening of the youngest women in our study (age 14 years in 2007). It is also possible that the intensive surveillance of the trial populations detected more non-HPV16/18 related CIN than occurs in real world screening programs, where many of these abnormalities will develop and resolve within the screening interval and are thus never observed. This could explain why a larger percentage of disease appears to be prevented in the population setting than in the trial setting. The predominance of HPV16 related disease in younger women has implications for the post-vaccination surveillance of high-grade lesions, as a greater impact of the vaccine may be expected initially. As vaccinated cohorts age and span a broader age range that includes women in their 30s, where other less oncogenic HPV types are more prevalent and may take longer to progress, the impact of vaccination on the overall CIN3+ burden may be relatively reduced [2,12].

We observed a significant impact of vaccination on LG cytological abnormalities that was similar in magnitude to that observed for HG cytological lesions. In addition, the effect of partial vaccination was significant for LG cytology, but not for HG cytology, although this may be due to the larger number of outcomes in this group. Because a greater proportion of HG cytology is due to vaccine-related HPV types than LG cytology, we would have expected to see a greater impact on HG as compared with LG cytological lesions. However, this result was sensitive to whether we counted person-time in between dose one to three for those women who ended up being fully vaccinated (see Additional file 1: Table S1). Abnormalities occurring during this period are likely to be a result of pre-existing HPV infection due to the short time frame between doses, but we believe that this reflects the real world vaccine effectiveness in the population. Two meta-analyses showed that the contribution of HPV16 and 18 worldwide in LSIL lesions is approximately 34% [8,23] while in CIN3, it is approximately 57% [23], with some regional variation. Recent Australian data show a relatively high proportion, approximately 41%, of LG cytological lesions contain HPV types 16/18/6/11 in women aged 25 years and under (personal communication JMLB) and this may also in part explain our observed reduction in LG cytological abnormalities.

There are several potential limitations of our analysis. We did not have a complete date of migration out of Victoria and there may be a small degree of loss to follow-up on the VCCR. The data linkage for this analysis was conducted according to accepted protocols; although some inaccuracies are an inherent part of the linkage methodology, the effect of this is likely to be small [24]. Any under-reporting of doses to the NHVPR given in the community to women who were within the school cohort, or doses given to women who opt-off the register, would have biased results towards the null if a proportion of unvaccinated women were truly vaccinated. Follow-up time was limited due to the recency of screening commencement among women vaccinated at school.

As this is an observational study, rather than a randomized study, women who chose to be vaccinated are likely to be different from those who are not vaccinated. Women of lower socioeconomic status were somewhat less likely to be vaccinated in our study, although results were unchanged in the adjusted analysis. Uncontrolled confounding by other factors may also have influenced the magnitude of the observed impact of the vaccine. However, as only women who had ever had a Pap test are included in this analysis, the effect of any sociodemographic factors that cause both lower vaccination coverage and screening uptake (for example, being a member of a culturally and linguistically diverse group) is minimized.

If fewer than three doses could provide sufficient protection against disease, vaccine programs could achieve higher coverage at a lower cost [25-27]. There are several possible explanations as to why we did not observe a protective effect of one or two doses. Notably, women who only received one or two doses of vaccine rather than the full course were younger at first screening (suggesting earlier sexual debut), older at vaccination and of lower socioeconomic status. Adjusting for age at first screening and SES may mitigate somewhat but cannot completely control for the underlying differences in this group of women, who appear to be more likely to have been infected prior to vaccination than the fully vaccinated group. Analysis including more women, vaccinated prior to sexual debut and taking into account underlying sociodemographic differences among women who do not complete the course, is needed to refine the estimates of impact against disease for partially vaccinated women.

Our results contribute to the growing body of evidence in Australia of a reduced burden of HPV-related disease since implementation of the vaccination program in 2007. Data from HPV prevalence studies show a significant reduction in the prevalence of vaccine HPV genotypes (6, 11, 16, 18) in a post-vaccination sample compared with a pre-vaccination sample and lower prevalence in both vaccinated and unvaccinated women compared with the pre-vaccine reference population [28]. A substantial impact of the HPV vaccine on genital warts has already been observed in Australia, with a decline by the end of 2009 of 59% in warts diagnosed at sexual health services among women in the target age group (26 and under in 2007), and a smaller decline of 28% among heterosexual men of similar age [29]. A similar decline in the incidence of genital warts has been reported in New Zealand, the United States and Sweden [30-33]. Ecologic data [34] and routine monitoring data [10,12] show significant declines in histologically confirmed high-grade cervical abnormalities since the Australian vaccination program commenced among women under 20 years of age. In 2011 we demonstrated an absolute decrease in incidence of high-grade cervical abnormalities in Victoria of 0.38% (CI 95% 0.61 to 0.16) for women under 18 years of age [34]. The main limitation of these analyses was that individual level data on vaccination status were not available and thus it was not possible to attribute the decline in disease solely to the vaccination program.

## Conclusions

In conclusion, we have demonstrated the first population level impact of a school-based HPV vaccination program on cervical abnormalities. The program included a two-year catch-up for girls aged 14- to 17-years and achieved high vaccination coverage rates. The impact of the vaccine was greatest for women completely vaccinated at younger ages and for the more serious cervical abnormalities CIN3/AIS, which are more likely to be due to the vaccine HPV types. The reduction in risk of cervical abnormalities was restricted to women who received three doses of vaccine; however, relatively few women received fewer than three doses and these women were older at vaccination than the other women. Over the next five years, the effect of the vaccine is expected to increase as cohorts of women vaccinated prior to sexual debut move through the screening program.

## Abbreviations

HG: High-grade; HPV: Human papillomavirus; HR: Hazard ratio; HSIL: High-grade squamous intraepithelial lesion; LG: Low-grade; LSIL: Low-grade squamous intraepithelial lesions; NHVPR: National HPV Vaccination Program Register; SEIFA: Socio-Economic Indexes for Areas; SES: Socioeconomic status; TVC: Total Vaccine Cohort; VCCR: Victorian Cervical Cytology Registry; VE: Vaccine effectiveness.

## Competing interests

DMG, JMLB and MS were investigators on an Australian Research Council Linkage grant, for which CSL biotherapies was a partner organization. JMLB has been an investigator on investigator designed unrestricted epidemiological research grants partially funded through bioCSL but received no personal financial benefits.

## Authors’ contributions

MS, DMG and JMLB were principal investigators of the study, with assistance from KD and GC. JMLB, DMG and MS designed the study. AB conducted the statistical analysis and interpretation of results was led by DMG, JMLB and MS. DMG led the writing of the manuscript, together with AB, JMLB and MS, and all authors contributed to the final report. All authors read and approved the final manuscript.

## Pre-publication history

The pre-publication history for this paper can be accessed here:

http://www.biomedcentral.com/1741-7015/11/227/prepub

## Supplementary Material

Additional file 1: Table S1 Rate of histological and cytological cervical abnormalities for completely vaccinated, partially vaccinated and unvaccinated women using final dose status method (secondary analysis using ‘efficacy’ method). Click here for file
