# Supplementary material for: Impact of a population-based HPV vaccination program on cervical abnormalities: a data linkage study
Source: BMC Med. 2013 Oct 22;11:227. doi: 10.1186/1741-7015-11-227 (PMC4015688; doi:10.1186/1741-7015-11-227)
Supplement: Additional file 1: Table S1 — Rate of histological and cytological cervical abnormalities for completely vaccinated, partially vaccinated and unvaccinated women using final dose status method (secondary analysis using ‘efficacy’ method). [file 1741-7015-11-227-S1.docx]

**Table S1: Rate of histological and cytological cervical abnormalities for completely vaccinated, partially vaccinated and unvaccinated women using final dose status method (secondary analysis using ‘efficacy’ method)**

| **Outcome** |  | **Hazard ratio** |
| --- | --- | --- |
| **Histological abnormalities** |  |  |
| Any high grade | Unvaccinated | 1.0 |
|  | Vaccinated (unadjusted) | 0.73 (0.58–0.92) |
|  | Vaccinated (adjusted) | 0.70 (0.55–0.88) |
|  | *1 dose* | *1.26 (0.78–2.04)* |
|  | *2 doses* | *1.02 (0.66–1.58)* |
|  | *Complete* | *0.62 (0.48–0.79)* |
|  | *1 or 2 doses* | *1.11 (0.79–1.57)* |
|  | *2 or 3 doses* | *0.66 (0.52–0.83)* |
| CIN3/AIS | Unvaccinated | 1.0 |
|  | Vaccinated (unadjusted) | 0.68 (0.48–0.97) |
|  | Vaccinated (adjusted) | 0.64 (0.45–0.92) |
|  | *1 dose* | *1.59 (0.83–3.03)* |
|  | *2 doses* | *0.87 (0.43–1.75)* |
|  | *Complete* | *0.54 (0.36–0.79)* |
|  | *1 or 2 doses* | *1.16 (0.69–1.93)* |
|  | *2 or 3 doses* | *0.57 (0.39–0.83)* |
| CIN2 | Unvaccinated | 1.0 |
|  | Vaccinated (unadjusted) | 0.76 (0.57–1.01) |
|  | Vaccinated (adjusted) | 0.73 (0.55–0.97) |
|  | *1 dose* | *0.89 (0.45–1.76)* |
|  | *2 doses* | *1.03 (0.60–1.75)* |
|  | *Complete* | *0.68 (0.51–0.92)* |
|  | *1 or 2 doses* | *0.97 (0.62–1.52)* |
|  | *2 or 3 doses* | *0.72 (0.54–0.96)* |
| CIN1 | Unvaccinated | 1.0 |
|  | Vaccinated (unadjusted) | 0.87 (0.71–1.07) |
|  | Vaccinated (adjusted) | 0.85 (0.69–1.04) |
|  | *1 dose* | *0.97 (0.59–1.58)* |
|  | *2 doses* | *0.92 (0.60–1.39)* |
|  | *Complete* | *0.83 (0.67–1.02)* |
|  | *1 or 2 doses* | *0.94 (0.67–1.31)* |
|  | *2 or 3 doses* | *0.84 (0.68–1.03)* |
|  |  |  |
| **Cytological abnormalities** |  |  |
| High-grade cytology | Unvaccinated | 1.0 |
|  | Vaccinated (unadjusted) | 0.78 (0.67–0.90) |
|  | Vaccinated (adjusted) | 0.76 (0.65–0.88) |
|  | *1 dose* | *0.94 (0.66–1.35)* |
|  | *2 doses* | *1.04 (0.78–1.38)* |
|  | *Complete* | *0.71 (0.61–0.83)* |
|  | *1 or 2 doses* | *1.00 (0.79–1.27)* |
|  | *2 or 3 doses* | *0.74 (0.64–0.86)* |
| Low-grade cytology | Unvaccinated | 1.0 |
|  | Vaccinated (unadjusted) | 0.82 (0.77–0.86) |
|  | Vaccinated (adjusted) | 0.81 (0.76–0.85) |
|  | *1 dose* | *0.92 (0.80–1.06)* |
|  | *2 doses* | *0.77 (0.68–0.87)* |
|  | *Complete* | *0.80 (0.76–0.85)* |
|  | *1 or 2 doses* | *0.83 (0.75–0.91)* |
|  | *2 or 3 doses* | *0.80 (0.75–0.84)* |

Footnotes

*Rate per 1,000 person-years

All high grade histology defined as CIN2, CIN3, AIS, and mixed CIN3/AIS

High-grade cytology defined as possible high-grade squamous intraepithelial lesion (HSIL), HSIL, HSIL with possible microinvasion/invasion, squamous cell carcinoma, possible high-grade endocervical glandular lesion, AIS, AIS with possible microinvasion/invasion and adenocarcinoma

Low-grade cytology defined as possible low-grade squamous intraepithelial lesions (LSIL), LSIL, and atypical endocervical cells of uncertain significance

Unvaccinated refers to women screened who did not receive any dose of HPV vaccine, completely vaccinated refers to women who were clinically completely vaccinated with 3 doses of HPV vaccine

Hazard ratios adjusted for remoteness, SES and age at first screen.
